# Identification and validation of reference genes for RT-qPCR normalization in wheat meiosis

**DOI:** 10.1038/s41598-020-59580-5

**Published:** 2020-02-17

**Authors:** José Garrido, Miguel Aguilar, Pilar Prieto

**Affiliations:** 1 Plant Breeding Department, Institute for Sustainable Agriculture, Agencia Estatal Consejo Superior de Investigaciones Científicas (CSIC), Alameda del Obispo s/n, Apartado 4084, 14080 Córdoba, Spain; 2 Área de Fisiología Vegetal. Universidad de Córdoba. Campus de Rabanales, edif. C4, 3ª planta, Córdoba, Spain

**Keywords:** Gene expression, Reverse transcription polymerase chain reaction, Plant breeding

## Abstract

Meiosis is a specialized type of cell division occurring in sexually reproducing organisms to generate haploid cells known as gametes. In flowering plants, male gametes are produced in anthers, being encased in pollen grains. Understanding the genetic regulation of meiosis key events such as chromosome recognition and pairing, synapsis and recombination, is needed to manipulate chromosome associations for breeding purposes, particularly in important cereal crops like wheat. Reverse transcription-quantitative PCR (RT-qPCR) is widely used to analyse gene expression and to validate the results obtained by other transcriptomic analyses, like RNA-seq. Selection and validation of appropriate reference genes for RT-qPCR normalization is essential to obtain reproducible and accurate expression data. In this work, twelve candidate reference genes were evaluated using the mainstream algorithms geNorm, Normfinder, BestKeeper and ΔCt, then ranked from most to least suitable for normalization with RefFinder. Different sets of reference genes were recommended to normalize gene expression data in anther meiosis of bread and durum wheat, their corresponding genotypes in the absence of the *Ph1* locus and for comparative studies among wheat genotypes. Comparisons between meiotic (anthers) and somatic (leaves and roots) wheat tissues were also carried out. To the best of our knowledge, our study provides the first comprehensive list of reference genes for robust RT-qPCR normalization to study differentially expressed genes during male meiosis in wheat in a breeding framework.

## Introduction

The study of biological processes usually involves gene expression analyses and quantification. Reverse transcription-quantitative polymerase chain reaction (RT-qPCR) is the most widely used technique nowadays to analyse gene expression due to factors like cost-effectiveness, specificity and sensitivity^1^. However, to achieve accurate and reliable results, sample-to-sample variation and experimental error need to be controlled by making use of normalization strategies^2,3^. The most common and effective method for RT-qPCR normalization is the use of reference genes (RGs), often referred as control genes or housekeeping genes, as internal controls. RGs need to be validated for a given experimental setup, since there are no universal RGs suitable for every tissue or experimental condition as the vast scientific literature on this topic proves. Validation is a critical step, since the use of random, putative or unvalidated RGs introduces significant biases in results^1,4^.

The main premise for RGs is that their expression remains unchanged or relatively invariant in the experimental context under study, which is not always the case in practice^1^. Thus, the available validation methods perform a selection based on the expression stability of the candidate RGs. That is to say, the least variable genes are the most stably expressed and the most suitable for normalization. Among the available RG methods, the most frequently used are geNorm^3^, or its updated version, qBase^5^, BestKeeper^2^ and Normfinder^6^. geNorm performs pairwise comparisons, calculating the gene stability value (M) as the mean standard deviation of the log-transformed expression ratios for every candidate RG. Moreover, given that several RGs must be used for accurate normalization, geNorm calculates the pairwise variation (V_n/n+1_) on the normalization factor (NF_n_/NF_n+1_) resulting from the inclusion of additional RGs, in order to estimate the optimal number of RGs needed for normalization. However, because the method also top ranks the candidate RGs with high similarity in their expression profiles, it is vulnerable to recommend co-regulated RGs^6^. Another validation algorithm, NormFinder, uses a model-based approach to calculate a stability value that ranks RGs according to their intra- and inter-group variation, thus being less prone to selection of co-regulated RGs. But, unlike geNorm, sample size affects the robustness of this method^7^. BestKeeper uses the raw quantitation cycle (Cq) values of each RG and employs pairwise correlation analysis (Pearson correlation coefficient) to rank RGs. However, as addressed by the authors, the method assumes data normality and homogeneity of variances. Otherwise, the use of the Pearson correlation coefficient would be invalid. Another popular validation method is the comparative ΔCt method^8^, which ranks RGs by their mean standard deviation in pairwise comparisons (ΔCq).

As results of the different strategies used to calculate the RG stability values, each algorithm may rank differently the same RG. Each validation method has strengths and limitations, so a comprehensive consensus among them may counteract any bias and assure the selection of the best RGs. RefFinder^9^ is a web-based tool which integrates the candidate RGs validation methods described above and generates a comprehensive final weighed rank list by geometric averaging the RGs rankings given by the different validation methods. It has been used in numerous research projects due to user-friendly interface and fast results.

Wheat is one of the most important food crops worldwide, with more than 218 Mha cultivated and a production exceeding 771Mt in 2017 (http://faostat.fao.org). The study of its genome organization (allohexaploid; AABBDD; 2n = 6 x = 42) is necessary for geneticists and plant breeders. Particularly, the knowledge about how homologous chromosomes (equivalent chromosomes from the same subgenome) specifically identify each other to associate properly in pairs at the beginning of meiosis, is essential in a plant breeding framework^10,11^.

In polyploidy species like wheat, meiosis must be smartly regulated. Each chromosome needs to identify the right partner to correctly associate in pairs, what means that, despite genome complexity (hexaploid wheat has **A**, **B** and **D** subgenomes and tetraploid wheat has **A** and **B** subgenomes) polyploid wheat behave as diploid during meiosis. Thus, at the beginning of meiosis, only homologous chromosomes correctly associate in pairs and achieve recombination. For example, chromosome **1 A** only associates correctly and recombines with chromosome **1 A** and not with the homoeologous (similar chromosome from the related subgenomes) chromosomes **1B** or **1D**. The accuracy and efficiency of the mechanisms that allow correct chromosome associations during meiosis have a big effect on the fertility of wheat plants. In contrast, this great genome stability prevents pairing and recombination between wheat chromosomes and those from related species, having negative effects for plant breeding purposes. In wheat, the *Ph1* locus suppresses recombination between homoeologous chromosomes^12–15^, and has been recently associated with the *TaZYP4-B2* gene^16,17^. In the absence of the *Ph1* locus, recombination is possible between the homoeologous chromosomes of wheat or between those of wheat and other species^18^. Thus, understanding the molecular basis of chromosome recognition, pairing and recombination during meiosis in wheat can contribute to provide useful tools to manipulate chromosome associations in the context of breeding, and therefore, facilitate the transfer of desirable agronomic traits from related species into wheat^10,19^.

Much information about the processes involved in the synaptonemal complex formation, recombination and chromosome segregation during meiosis is available, but very little is known about how chromosomes precisely identify a partner to correctly associate in pairs to further recombine and successfully segregate. Chromosome recognition and pairing are extremely dynamic processes, which occur only between some regions of the chromosomes in a non-synchronized way from one nucleus to the other, increasing the difficulties to study the process profoundly^20^.

Recently, the reference genome of hexaploid wheat has been made available, having 21 chromosome-like sequence assemblies annotated with 107,891 high-confidence genes^21^. The availability of a reference genome greatly facilitates functional studies and can be used as a tool to study the DNA sequences that might play a role in the processes occurring during early meiosis and the proteins interacting with them.

The aim of this work was the identification of reliable RGs to allow accurate measurements for gene expression analysis in genomic studies and unravelling the regulation of different processes occurring during meiosis in wheat. We have validated specific sets of RGs suitable for expression studies developed in wheat anther in premeiosis and at different stages of meiosis. Hexaploid and tetraploid wheat were used in this study, both in the presence and in the absence of the *Ph1* locus. Comparative studies with somatic tissues are also described.

## Materials and Methods

### Plant material

Meiotic anthers and somatic tissues were isolated form hexaploid (bread) wheat, *Triticum aestivum* L., cv. Chinese Spring (CS) and the *ph1b* mutant^14^, as well as tetraploid (durum) wheat (*Triticum turgidum* L. ssp. *Durum*, cv. Senatore Cappelli and the corresponding *ph1* mutant, DES35^22^. All wheat lines were kindly provided by Dr. Steve Reader from John Innes Centre (Norwich, U.K.).

Seeds were germinated in the dark at 25 °C on wet filter paper in Petri dishes for 2 days and then transferred to pots and grown in the greenhouse at 24 ± 2 °C with a 16/8 h photoperiod.

One anther per floret was carefully checked in order to determine the meiosis stage as previously described^23^. We collected the two remaining anthers in premeiosis (PM), with visible sporogenous archesporial columns (SACs) but no signs of meiosis; prophase I (PRO), formed by an even mix of leptonema-zygonema, pachynema, and diplonema-diakinesis; telophase I to II (TT) mix of stages; and immature pollen (IP). Collected anthers were kept in ice-cold phosphate buffer saline. A mix of 25–30 anthers at the same meiotic stage collected from 3 different spikelets constituted a sample (biological replicate). Somatic cells from vegetative tissues, 2-week-old leaves (L) and 2 cm long root tips (R) from germinating seeds, were also collected for comparative studies. All samples were frozen in liquid nitrogen and stored at −80 °C until use.

### Microarray screening for candidate RGs and primer design

New meiosis-specific candidate RGs were selected using the previously published microarray data^23^. Raw data were downloaded from the GEO database (Accession: GSE6027) and analyzed using Arraystar (version 15.3.a DNASTAR. Madison, WI). Raw expression intensities were normalized through the Robust Multichip Average (RMA) method. Moderated t test and false discovery rate (FDR) for multiple testing corrections, were used with an adjusted P < 0.05. The wheat consensus sequences used for the Affymetrix GeneChip Wheat Genome Array (Affymetrix, CA, USA) design were downloaded and BLASTed against the IWGSC RefSeq annotation v1.1 in The European UseGalaxy server (https://usegalaxy.eu/)^24–26^, in order to find the updated annotations of the represented genes. Additionally, the SwissProt ID was used to confirm the IWGSC gene IDs in Uniprot^27^ and select defined loci in case of big gene families. Microarray transcripts showing stable expression along the meiosis stages were selected. Specific primer pairs were designed in Primer-BLAST^28^ to yield amplicon of preferred sizes ranging 120–200 bp, using *Aegilops tauschii* for target specificity. Candidate oligos were then confirmed to anneal on all the homoeologous loci by BLASTN search (http://plants.ensembl.org/Triticum_aestivum/Tools/Blast) and visual inspection on the predicted gene models and RNA-seq mapped transcripts, represented in JBrowse (https://urgi.versailles.inra.fr/jbrowseiwgsc/gmod_jbrowse/?data=myData%2FIWGSC_RefSeq_v1.0&loc=chr1A%3A1.499351&tracks=DNA%2CHighConfidenceGenesv1.1%2CRNASeqDong%2CRNASeqNRGene&highlight=), using the sequence search track feature for primer mapping. Optimal primer concentration and annealing temperatures were determined using a gradient RT-qPCR. The specificity of the primers was verified by agarose gel electrophoresis and melting curves showing single amplicons.

### Gene duplication analysis

Gene duplication events affecting the RGs were analysed in the CoGe web platform (https://genomevolution.org/coge/) using SynMap2^29^. The results can be regenerated and the data downloaded for further evaluation at https://genomevolution.org/r/17850 (persistent link).

### RNA extraction and cDNA synthesis

Frozen tissues (100 mg) were placed in pre-chilled 2.0 mL RNase-free microcentrifuge tubes, containing two (DEPC-treated) 3 mm stainless steel balls and frozen in liquid nitrogen, then grinded to fine powder in a Retsch Mixer Mill, model MM 301 (Retsch GmbH, Germany) at 25 Hz for 30 seconds. Total RNA was extracted from different tissues using Direct-zol RNA MiniPrep Kit (Cat. R2051, Zymo Research, Irvine, CA.) and treated with RNAse-free DNAse according to the manufacturer’s manual. Residual DNA contamination was checked by PCR. Purified RNA was quantified using a NanoDrop ND-1000 Spectrophotometer (NanoDrop Technologies, Wilmington, DE, USA) and the RNA integrity assessed by agarose gel electrophoresis. First-strand cDNA synthesis was carried out with the iScript cDNA Synthesis kit (Bio-Rad Laboratories, Hercules, CA), using 1 µg of purified total RNA per 20 µL of reaction volume. All cDNAs were diluted 5-fold with nuclease-free water prior to being used in the qPCR step.

### RT-qPCR

RT-qPCR runs were performed in CFX Connect Real-Time PCR Detection System (Bio-Rad). One µL of cDNA was added to each PCR reaction mix (20 µL), containing 0.25 µM of each primer and 10 µL of 2X iTaq SYBR Green supermix (Bio-Rad Laboratories, Hercules, CA). The following protocol was used: an initial enzyme activation/cDNA denaturation step at 95 °C for 1 min, followed by 40 cycles at 95 °C for 15 sec, 60 °C for 15 sec and 72 °C for 15 sec, with a final standard dissociation protocol to obtain the melting profiles. Data were acquired using the CFX Manager software.

### Data analysis

Mean PCR efficiencies were calculated by LinRegPCR, version 2018.0^30^. Expression stability of the candidate RGs was evaluated using RefFinder^9^ (https://github.com/fulxie/RefFinder), which integrates the algorithms geNorm, Normfinder and BestKeeper, as well as the comparative ΔCt method^2,3,6,8^. The efficiency-corrected Cq (CqE) was calculated according to the formula

$$CqE=Cq(\log (E)/\log (2))$$

and used as input to calculate the stability values by the geNorm and NormFinder algorithms. A comprehensive, weighted ranking of the RGs for each experimental condition was generated by calculating the geometric mean of the rank values gathered by each gene in the different algorithms. The pairwise variation (V) used to determine the optimal number of RGs was calculated separately using the geNorm algorithm. Relative fold change values of the wheat *Rec8* gene, as expression ratio between the different samples and PM, were calculated using the resulting normalization factor (NF) of the selected RGs^5^.

Data were analysed in Statistix 10. Shapiro-Wilk test (α = 0.05) was used to check data normality. One-way ANOVA, followed by Tukey HSD for multiple pairwise comparisons, or Dunnett’s test (two-tailed) for sample comparisons with PM as control treatment (α = 0.05) were applied. Three biological samples and two technical replicates were analysed. Means, standard errors and statistical significances for each sample were represented in figures. Tukey HSD results were displayed as letters: means sharing a common letter were not significantly different. Dunnett’s test results were displayed as asterisks (*P < 0.05, **P < 0.01).

## Results

### Selection of candidate RGs

**Figure 1:**
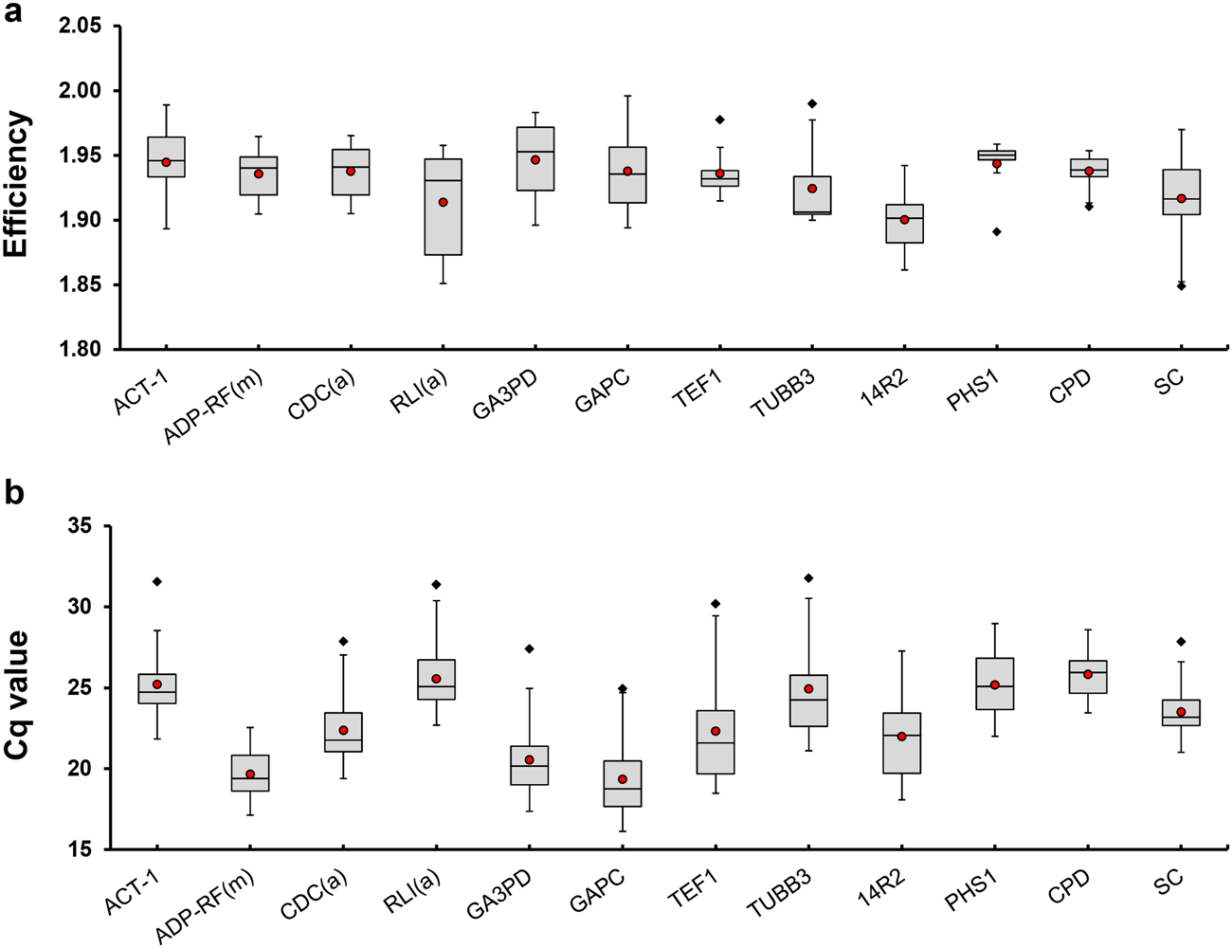
Expression parameters of the 12 candidate RGs used in this study. The efficiency values obtained for all the samples (**a**) and Cq values obtained in anthers undergoing meiosis and in somatic tissues (**b**). Box plots represent the interquartile range (IQR, 25^th^–75^th^). Horizontal bars and red dots represent the median and mean, respectively. Whiskers indicate the minimum and maximum values. Black diamonds represent outliers, values smaller or larger than 1.5 times the IQR. Coefficients of variation are annotated above the plots.

With the aim of finding candidate RGs having stable expression along the different meiosis stages we reanalysed a previously published wheat meiosis microarray^23^. Gene correspondences between consensus transcripts, used for the Affymetrix GeneChip Wheat Genome Array design, and the current bread wheat annotation (IWGSC RefSeq v1.1) were set using BLASTN. The analysis of the overall gene expression variation (Table 1) reveals that most of the significant fold change variation at 2-fold level and above took place between anthers in PM and mature anthers (MAN). Only few hundreds were differentially expressed during meiosis, which means that a considerable number of genes remained potentially stably expressed. This is consistent with the results of Martín and colleagues^31^, who have found that only a small fraction of genes were differentially expressed at early meiosis. Several potential candidate RGs were identified and specific primers were designed for RT-qPCR. The final selected candidate RGs and primer pairs (Table 2 and Supplementary Table S1) seemed to be stably expressed across meiosis (Supplementary Table S2) and yield single amplicons in all the genotypes tested (Supplementary Figure S1), with sizes suitable for RT-qPCR between 77 and 276 bp. Besides, they showed high PCR mean efficiencies in all the tissue samples tested, ranging from 1.900 to 1.946 (R^2^ > 0.99). On the other hand, the Cq values ranged from 20 to 30, approximately (Supplementary Data S1 and Fig. 1), with the leaf samples showing higher Cq values as average than anthers and roots.

**Table 1:** Affymetrix GeneChip Wheat Genome Array analysis indicating the number of transcripts at or above 2-fold change per comparison and confidence level. PM = premeiosis; LP = leptotene to pachytene; DA = diplotene to anaphase I; TT = telophase I to telophase II; T = tetrads; IP = immature pollen; MAN = mature anthers.

Comparison	Confidence			R^2^
	90%	95%	99%	
**PM vs MAN**	14582	14581	14574	0.6851
**PM vs LP**	203	179	113	0.9906
**PM vs DA**	300	298	279	0.9912
**PM vs TT**	514	512	493	0.9864
**PM vs T**	1497	1304	631	0.9654
**PM vs IP**	0	0	0	0.9632

**Table 2:** Description of the selected candidate RGs.

Gene/primer symbol	IWGSC RefSeq v1.1 ID	Description
**14R2**	TraesCS4D02G046400 TraesCS4A02G268700 TraesCS4B02G045500	14-3-3 protein
**ACT-1**	TraesCS1A02G020500 TraesCS1B02G024500 TraesCS1D02G020000	Actin
**ADP-RF(m)**	TraesCS5A02G467400 TraesCS5B02G479100 TraesCS5D02G480200 TraesCS1D02G305900 TraesCS1A02G306200 TraesCS1B02G317000 TraesCS3D02G330500 TraesCS3A02G337300 TraesCS3B02G368600	ADP-ribosylation factor
**CDC(a)**	TraesCS4D02G267800 TraesCS4B02G268400 TraesCS4A02G035700 TraesCS4D02G267600 TraesCS4B02G268200 TraesCS4A02G035500	Cell division control protein
**CPD**	TraesCS1B02G351000 TraesCS1A02G338500 TraesCS1D02G340700	Cyclic phosphodiesterase-like protein
**GA3PD**	TraesCS7A02G313100 TraesCS7B02G213300 TraesCS7D02G309500	Glyceraldehyde-3-phosphate dehydrogenase
**GAPC**	TraesCS6D02G196300 TraesCS6A02G213700 TraesCS6B02G243700	Glyceraldehyde-3-phosphate dehydrogenase
**PHS1**	TraesCS7B02G132000 TraesCS7A02G233700 TraesCS7D02G233900	Poor homologous synapsis 1 protein
**RLI(a)**	TraesCS4A02G143000 TraesCS4B02G160000 TraesCS4D02G159400	RNase L inhibitor-like protein
**SC**	TraesCS5B02G233800 TraesCS5D02G242500 TraesCS5A02G235400	Salt tolerant protein
**TEF1**	TraesCS4A02G107700 TraesCS4A02G107600 TraesCS4B02G196800 TraesCS4B02G196900 TraesCS4D02G197100 TraesCS4D02G197200	Elongation factor 1 alpha-subunit
**TUBB3**	TraesCS6D02G130500 TraesCS6B02G169300 TraesCS6A02G192000LC	Beta-tubulin 3

Some of the candidate RGs selected belong to gene families commonly used to normalize gene expression in several species, tissues and experimental conditions, such as *Glyceraldehyde-3-phosphate dehydrogenase* (GA3PD and GAPC), *Elongation factor-1α* (TEF1), *Actin* (ACT-1), and *Tubulin* (TUBB3). We have redesigned primers for two genes proposed previously in wheat meiosis to be included in our study: *Flat gene 2*, identified here as *14R2* (14-3-3 protein coding), and *PHS1*^23^. In addition, three previously validated RGs for RT-qPCR in wheat tissues were also tested: *ADP-ribosylation factor* (ADP-RF(m)), *RNase L inhibitor-like* (RLI(a)) and *Cell division control protein* (CDC(a))^32,33^. Finally, two new potential candidate RGs were identified in our study: an uncharacterized *cyclic phosphodiesterase*-like gene (CPD) and the wheat *Salt tolerant protein* gene (SC)^34^. The B homoeolog of the SC gene (TraesCS5B02G233800) locates within the *Ph1* locus^35^, therefore it is not expressed in *ph1b* mutant genotypes (CS*ph1* and DES35).

Gene duplication analysis revealed that three RGs (*Elongation factor-1α*, *ADP-ribosylation factor* and *Cell division control protein*) have collinear paralogs sharing high identity (Supplementary Table S3), which might be amplified by the respective primers pairs, if expressed.

### Analysis of gene expression stability in different wheat genotypes and tissues

The expression stability of the candidate RGs was evaluated by RT-qPCR in the different tissues and the results were analyzed using standard validation methods and algorithms: the comparative ΔCt method, geNorm, Normfinder and BestKeeper, integrated in RefFinder. The stability of the candidate RG measures were calculated by each algorithm and ranked accordingly from the most to the least suitable gene to be chosen as reference for RT-qPCR normalization (Supplementary Tables S4 and S5). Each method set different gene rankings for a given comparison, although they often concur in sorting approximately the same candidate RGs at the top (most stable) or low (least stable) ends of the lists. RefFinder generates a final weighed rank list for every comparison by geometric averaging the rankings achieved by the entire candidate RGs across the different validation methods (Table 3). Overall, it is found that the three most frequently top ranked genes are RLI(a), ADP-RF(m) and CPD for anthers during meiosis, and RLI(a), ACT-1 and SC for comparisons containing anthers in meiosis and somatic tissues. Therefore, at least one of them is found among the top three recommended genes in any experimental situation. On the other hand, the most frequent bottom ranked genes were TUBB3 and TEF1, adding 14R2 for anthers during meiosis, hence they are rarely included among the final recommended genes.

**Table 3:** RefFinder expression stability weighed ranking for the twelve RGs.

Group	Rank	CS	CS*ph1*	Cappelli	DES35	CS/CS*ph1*	Cappelli/DES35	CS/Cappelli	CS*ph1*/DES35	All genotypes
**MEIOSIS**	1	RLI(a)	TEF1	ADP-RF(m)	RLI(a)	CPD	RLI(a)	ADP-RF(m)	TEF1	CPD
	2	CPD	CPD	RLI(a)	ADP-RF(m)	PHS1	GA3PD	CPD	RLI(a)	ADP-RF(m)
	3	ADP-RF(m)	SC	CDC(a)	14R2	TUBB3	ADP-RF(m)	RLI(a)	GA3PD	GA3PD
	4	SC	TUBB3	GA3PD	GAPC	SC	ACT-1	CDC(a)	TUBB3	RLI(a)
	5	CDC(a)	PHS1	GAPC	GA3PD	ACT-1	CDC(a)	SC	GAPC	CDC(a)
	6	PHS1	14R2	CPD	SC	GA3PD	14R2	ACT-1	SC	ACT-1
	7	ACT-1	GAPC	SC	CPD	GAPC	CPD	GA3PD	PHS1	SC
	8	14R2	RLI(a)	14R2	CDC(a)	TEF1	GAPC	TUBB3	CPD	TUBB3
	9	TEF1	GA3PD	ACT-1	ACT-1	RLI(a)	SC	TEF1	ACT-1	PHS1
	10	GAPC	ACT-1	TUBB3	TUBB3	ADP-RF(m)	TUBB3	PHS1	CDC(a)	TEF1
	11	GA3PD	ADP-RF(m)	TEF1	TEF1	CDC(a)	TEF1	14R2	ADP-RF(m)	GAPC
	12	TUBB3	CDC(a)	PHS1	PHS1	14R2	PHS1	GAPC	14R2	14R2
**MEIOSIS AND SOMATIC TISSUES**	1	RLI(a)	SC	14R2	14R2	SC	14R2	RLI(a)	ACT-1	ACT-1
	2	SC	CPD	RLI(a)	GA3PD	ACT-1	RLI(a)	ACT-1	RLI(a)	RLI(a)
	3	14R2	PHS1	ACT-1	RLI(a)	RLI(a)	ACT-1	CDC(a)	SC	CDC(a)
	4	ACT-1	ACT-1	GA3PD	CDC(a)	CPD	GA3PD	14R2	PHS1	SC
	5	PHS1	GAPC	SC	ACT-1	PHS1	CDC(a)	GA3PD	GAPC	GA3PD
	6	CPD	RLI(a)	CDC(a)	SC	ADP-RF(m)	SC	ADP-RF(m)	CPD	ADP-RF(m)
	7	TEF1	CDC(a)	ADP-RF(m)	GAPC	CDC(a)	ADP-RF(m)	CPD	ADP-RF(m)	CPD
	8	ADP-RF(m)	GA3PD	GAPC	ADP-RF(m)	GAPC	GAPC	SC	CDC(a)	GAPC
	9	GA3PD	ADP-RF(m)	CPD	PHS1	GA3PD	CPD	GAPC	GA3PD	PHS1
	10	CDC(a)	TUBB3	TUBB3	CPD	TUBB3	PHS1	TUBB3	14R2	14R2
	11	GAPC	TEF1	TEF1	TEF1	TEF1	TUBB3	TEF1	TEF1	TUBB3
	12	TUBB3	14R2	PHS1	TUBB3	14R2	TEF1	PHS1	TUBB3	TEF1

Although each gene position varies for every sample set, wheat tetraploid lines, both in the presence and in the absence of the *Ph1* locus, essentially showed the same order for each meiosis sample. In fact, the most recommended ADP-RF(m) and RLI(a) genes, as well as the least recommended TUBB3, TEF1 and PHS1 genes were ranked similarly, both in the presence and in the absence of the *Ph1* locus in tetraploid wheat. In contrast, differences were found in both, general ranking order and top/bottom ranked genes between the *Ph1* and *ph1* genotypes in hexaploid wheat. This suggests that *Ph1* locus seems to be affecting the RG expression stability differently in hexaploid than in tetraploid wheat during meiosis.

### Recommended number of RGs

The optimal number of RGs to calculate the normalization factor (NF) was determined by geNorm, calculating the pairwise variation V_n/n+1_ between two sequential normalization factors, NF_n_ and NF_n+1_, and taking 0.15 as the cut-off value^3^. Calculations were made using the weighed rank orders determined by RefFinder. Results are shown in Table 4, which summarizes the optimal number of RG to calculate the NF for every experimental condition.

**Table 4:** Optimal number of RGs for normalization by determining the pairwise variation (V).

Comparison		Pairwise variation									
		V_2/3_	V_3/4_	V_4/5_	V_5/6_	V_6/7_	V_7/8_	V_8/9_	V_9/10_	V_10/11_	V_11/12_
**MEIOSIS**	**CS**	0.1581	**0.1158**	0.1215	0.0799	0.1014	0.0796	0.0821	0.0872	0.0855	0.0830
	**CS*ph1***	0.2668	0.1555	**0.0802**	0.0863	0.0892	0.1320	0.0906	0.1092	0.1211	0.1092
	**Cappelli**	0.1999	**0.1339**	0.1028	0.0776	0.0675	0.0891	0.0754	0.0841	0.0847	0.0916
	**DES35**	0.2235	**0.1108**	0.1066	0.1525	0.0816	0.0790	0.0801	0.0733	0.0647	0.0650
	**CS/CS*ph1***	0.2822	0.3187	0.2368	0.1625	**0.1495**	0.1206	0.1448	0.1087	0.1123	0.1123
	**Cappelli/DES35**	**0.1308**	0.1540	0.1419	0.0875	0.0951	0.1050	0.0778	0.0898	0.0818	0.0801
	**CS/Cappelli**	0.2139	0.1613	**0.1174**	0.1420	0.1480	0.1271	0.1079	0.0855	0.0963	0.0877
	**CS*ph1*/DES35**	0.2459	0.1649	**0.1363**	0.1752	0.1073	0.0965	0.1086	0.1128	0.0828	0
	**All genotypes**	0.3280	0.2119	0.1658	**0.1205**	0.1202	0.1362	0.1144	0.1130	0.1049	0
**MEIOSIS AND SOMATIC TISSUES**	**CS**	0.3448	0.2070	0.2245	0.2087	0.1567	**0.1353**	0.0923	0.0983	0.0895	0
	**CS*ph1***	0.2113	0.2487	0.2010	0.1591	0.1570	**0.1445**	0.1354	0.1573	0.1570	0.1591
	**Cappelli**	0.2126	0.1785	**0**	0.1345	0.3981	0.2600	0.1759	0.1333	0.1674	0.1332
	**DES35**	0.2125	0.1894	**0.1141**	0.2187	0.1225	0.1258	0.1136	0	0	0.1329
	**CS/CS*ph1***	0.2343	0.3412	0.2142	0.1804	0.1519	**0**	0.2521	0.1289	0.1759	0.1573
	**Cappelli/DES35**	0.2022	0.1787	0.1501	**0**	0.3610	0.1384	0	0	0.1660	0
	**CS/Cappelli**	0.2340	0.2917	0.1761	0.2098	**0**	0.1587	0.1381	0	0.1366	0.1366
	**CS*ph1*/DES35**	0.3846	0.2383	0.2314	0.1826	**0.1409**	0.1256	0.1143	0	0.2140	0
	**All genotypes**	0.2552	0.2840	0.2286	0.1864	**0**	0	0.3103	0.1480	0.3091	0.2347

Cells in bold indicate the first V_n/n+1_ values under the 0.15 cutoff and thus unnecessary addition of n + 1 reference genes.

The number of RGs required for accurate gene normalization during meiosis is three or four when the four genotypes are analysed independently or comparisons between hexaploid and tetraploid wheat lines are made, either in the presence or in the absence of the *Ph1* (CS vs Cappelli and CS*ph1* vs DES35; Table 4). The number of RGs needed go up to five or six when all genotypes are compared simultaneously or when comparing hexaploid wheat in the presence and in the absence of the *Ph1* (CS vs CS*ph1*), respectively (Table 4).

When the two somatic tissues (roots and leaves) are included in the analysis, the recommended number of RGs increases in all the cases except for the tetraploid wheat lines, reaching up to seven in some comparisons (CS and CS*ph1*) (Table 4). These results reveal the differences in gene expression among such different (meiotic and somatic) tissues. Comparisons involving wheat hexaploid lines in the presence and in the absence of the *Ph1* locus (CS vs CS*ph1*) require in both experiments (including or not somatic tissue) more RGs than when comparing wheat tetraploids genotypes (Cappelli vs DES35). Moreover, the *Ph1* locus does not seem to affect the number of recommended RG in tetraploid wheat for meiosis samples, either when somatic tissues are also considered. Our results suggest that the presence of D subgenome has a more relevant effect than the presence/ausence of *Ph1* locus on the pairwise variation (V) and on the number of RGs required for calculating the optimal NF, when the somatic tissues are included in the study. This may be explained by the dominance of the D subgenome over A and B in wheat (D > A > B)^36,37^, being the most dominantly expressed but with small differences among meiotic anthers, leaves and roots in hexaploid wheat^38^.

### Validation

**Figure 2:**
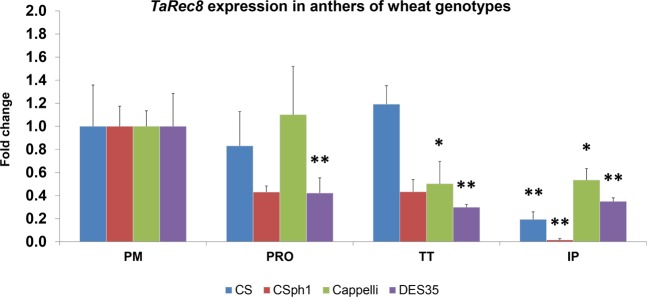
Expression profiling of *TaRec8* along meiosis stages in hexaploid and tetraploid wheat, including *ph1* mutants. PM: premeiosis; PRO: prophase I; TT: telophase I to II; IP: immature pollen. Means and standard error bars are represented. *P < 0.05, **P < 0.01 (Dunnett’s test).

**Figure 3:**
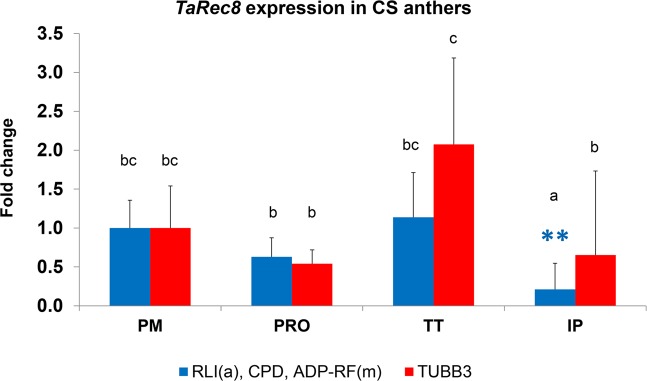
Example of the importance of using appropriated RGs. Differences in *TaRec8* expression in CS anthers along the different meiosis stages is illustrated using different RGs. Normalization was performed using both, the three more stable, recommended RGs (RLI(a), CPD and ADP-RF(m)) (represented in blue) and the least stable (TUBB3) represented in red. The relative expression values calculated in each case were significantly different for IP samples (Tukey HSD). The appropriated normalization method found the *TaRec8* fold change significantly lower in IP samples than PM, in contrast by using the least stable gene. Means followed by a common letter are not significantly different (Tukey HSD). **P < 0.01 (Dunnett’s test). PM: premeiosis; PRO: prophase I; TT: telophase I to II; IP: immature pollen.

In order to validate the selection of RGs and demonstrate their usefulness, we have performed an analysis, as an example, on the wheat *Rec8*-like meiotic cohesin expression^39^ in some of the different genotypes and tissues. A primer pair was designed specifically for RT-qPCR and to anneal in every homoeologous tetraploid and hexaploid wheat *Rec8*-like genes (Supplementary Table S1). The *TaRec8* expression in the four genotypes (CS, CS*ph1*, Cappelli and DES35) was normalized using the recommended gene set of CPD, ADP-RF(m), GA3PD, RLI(a) and CDC(a) (the five top-ranked RGs, see Tables 3 and 4). The results show some differences in *TaRec8* expression profiles among genotypes, but they share a general tendency to down regulation after prophase I, reaching the minimum in immature pollen cells (Fig. 2). This is coherent with previous studies showing protein expression at early meiosis and with the interaction of the protein with chromosomes in meiosis prophase I^39,40^. In addition, to illustrate the importance of choosing the appropriate RGs to study the expression of a specific gene, we compared the normalized expression of *TaRec8* in meiotic anthers of CS, calculated using the three recommended RGs (RLI(a), CPD and ADP-RF(m)), or the least recommended RG (TUBB3) for normalization. The results show significant differences in the relative quantification of IP samples in both cases (Tukey HSD) (Fig. 3), resulting in loss of statistical significance of the IP samples expression with respect to PM (Dunnett’s test) when the least recommended RG was used, highlighting the importance of using the validated recommended RGs set for a proper normalization.

**Figure 4:**
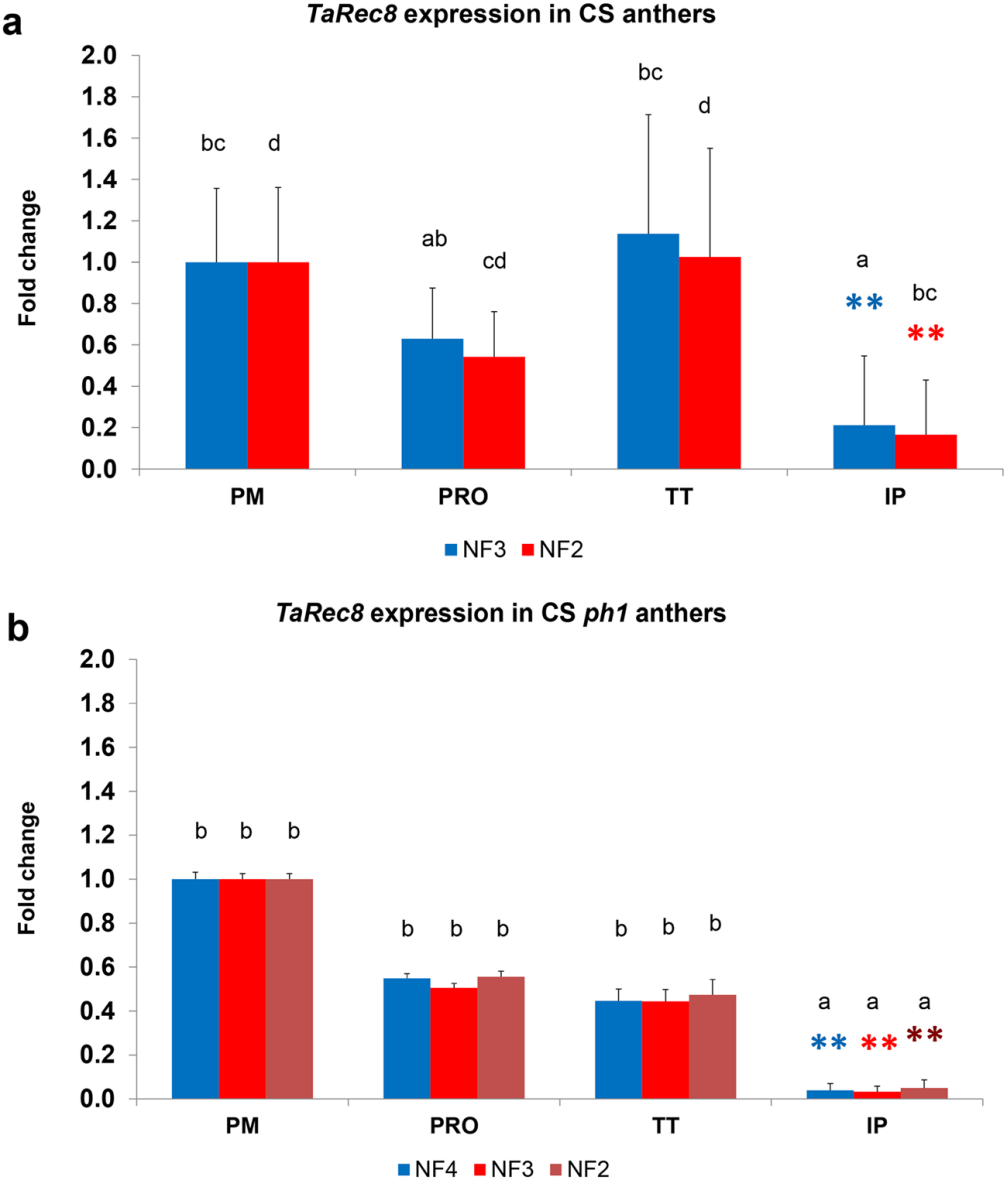
Effect of reducing the number of RGs to calculate the normalization factor. (**a**) *TaRec8* quantification along meiosis stages in CS anthers. Reduction from 3 to 2 RGs (NF3, NF2 respectively) causes significant underestimation in the relative expression, but small enough to yield the same data interpretation. (**b**) A further reduction is possible in the CS *ph1* mutant, from NF4 up to NF2 without significant differences in results. Means followed by a common letter are not significantly different (Tukey HSD). **P < 0.01 (Dunnett’s test). PM: premeiosis; PRO: prophase I; TT: telophase I to II; IP: immature pollen.

We have also explored the possibility of reducing the number of RGs in some analysis maintaining both accurate quantification and statistical significance, to avoid errors and misinterpretation of data because in some cases, the number of RGs needed went up to seven in some specific genotypes. So, in the case of the *TaRec8* gene, the reduction from three to two RGs can be done as the differences found for the IP samples with respect to any other stages (Tukey HSD and Dunnett’s) remain significant (Fig. 4A), although it causes small, but significant quantification changes in every meiosis stage (Tukey HSD). Thus, a relatively small loss of accuracy in the *TaRec8* expression quantification is observed, albeit the result’s interpretation is not altered. In the case of CS*ph1* genotype (Fig. 4B), the analysis of the meiosis normalization requires a recommended set of four RGs. A stepwise reduction to three or two RGs does not show significant differences in quantification with respect to the recommended four RGs, and at the same time, the significant differences observed in the relative expression of IP samples are retained.

**Figure 5:**
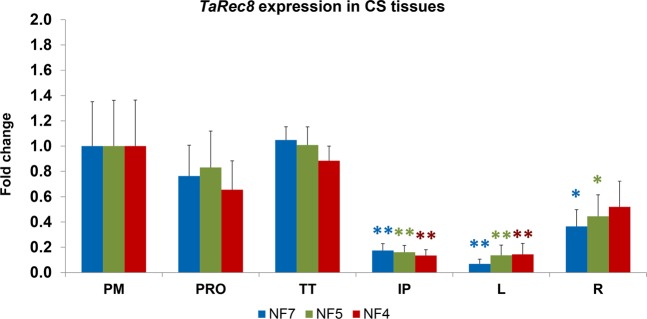
RGs reduction in *TaRec8* expression profiling in CS meiotic anthers and somatic tissues (leaves and root tips). Normalization factor was calculated using 7 (NF7), 5 (NF5) or 4 (NF4) reference genes. Reduction to NF5 is not significantly different from using NF7 and yields the same results. A further reduction to NF4 changes R sample quantification, which is no longer significantly lower than PM sample (see text for details). *P < 0.05, **P < 0.01 (Dunnett’s test). PM: premeiosis; PRO: prophase I; TT: telophase I to II; IP: immature pollen, L: leaves, R: root tips.

For some other cases, a gene reduction may be possible but to a lesser extent. For example, in the case of *TaRec8* expression in meiotic and somatic tissues, a stepwise reduction analysis from seven RGs down to the last two top-ranked RG was applied. The reduction from seven RGs to four makes significant differences in the *TaRec8* expression in root tips (somatic tissue), while no significant differences were detected down to three or two RGs in leaves (somatic) and meiosis samples, respectively (Supplementary Table S6). Therefore, the number of RGs can only be reduced to five to ensure an accurate *TaRec8* quantification in all the samples, while Dunnett’s test results provide the same statistical significances in both calculations. A further reduction to four RGs would not be possible as it caused enough data variation to significantly change the *TaRec8* quantification in root samples and modify the correct interpretation of the data (Fig. 5).

## Discussion

Although several validated RGs are available for expression data normalization in different somatic tissues in wheat^32,33,41^, to the best of our knowledge, none of them has covered the meiosis before. Moreover, similar analyses to find and validate suitable RGs for plant meiosis gene expression studies have been only conducted in rice so far^42^.

Selection of quality RGs suitable for robust normalization in wheat meiosis is challenging, especially due to the limited data available and the difficulty of collecting a good number of anthers in each specific stage of meiosis. In fact, for some wheat genotypes as those carrying the *Ph1* deletion, meiosis is not synchronized^43^, making the identification of each meiosis stage even more complicated. In addition, and as far as we know, although there are some massive approaches to study meiosis using transcriptomics and proteomics in cereals like maize^44–46^, rice^47,48^ and other plants^49^, the only massive transcriptomic study covering the whole meiosis process in wheat anthers was performed by Crismani and colleagues^23^, using the Affymetrix GeneChip Wheat Genome Array. Recent studies have examined gene expression in wheat meiotic anthers using RNA-seq^31,38^. However, these studies were restricted to early meiosis. Therefore, we decided to reanalyse the data from this microarray in order to find stably expressed transcripts during wheat meiosis.

Some of the selected candidates belong to *Glyceraldehyde-3-phosphate dehydrogenase*, *Elongation factor-1α*, *Actin*, and *Tubulin* gene families, which have been used and validated as suitable RGs in multiple studies^50^. Each of these RGs, however, has dozens of putative members in wheat, as revealed just by doing quick searches for the PFAM specific terms within the wheat transcriptome (http://plants.ensembl.org/Triticum_aestivum/Info/Index). Therefore, the microarray analysis helped to specifically identify candidate loci that are stably expressed during meiosis in wheat. Additionally, we validated two RGs previously proposed, 14R2 and PHS1^10^, using new updated primers specifically optimized for RT-qPCR. Another genes previously validated in somatic tissues, such as ADP-RF(m), RLI(a) and CDC(a)^32,33^, were found potentially promising in our preliminary screening. Finally, two new RGs, CPD and SC, were also identified as potentially not regulated during meiosis.

We have investigated the existence of gene duplicates for our candidate RGs, in order to determine if primers could also detect their expression. Thus, we have found that TEF1 and CDC(a) can anneal also to tandemly duplicated paralogs of these RGs located in their proximity, and ADP-RF(m) can detect gene expression of collinear segmental duplicates from wheat chromosomes 1, 3 and 5 (Table 2). All genes mentioned share high CDS identity and primers yield same size amplicons. Re-designing of specific primers for one or another homoeologous group is difficult if not virtually impossible, since main differences rely on some SNPs found along the coding sequence. An alternative would be to design primers for the more divergent 5′ and 3′ UTR regions. However, this would be useful mostly to study expression differences within the same species, since it is very unlikely that UTRs from orthologous genes between different species share enough sequence homology to be amplified by a common set of primers, as well as avoid the in- and out-paralogs amplification. Besides, such UTR regions are not characterized for every gene in the current wheat annotation.

As discussed in some papers, this is not a potential problem. For example, Brunner and colleagues^51^ propose that amplification of multiple family members might result in a more stable internal control than single gene amplification. Although paralogous genes might have different stability and expression profiles, the wheat microarray expression data suggest that expression of the TEF1, CDC and ADP-RF paralogous genes are similar across meiosis (except for CDC, since specific probes for the Ta.54227 unigene could not were identified). On the other hand, their expression stability was ranked by the different validation algorithms and, in fact, these RGs are among the most recommended in some analyses.

Our study covers important genotypes and relevant samples comparisons for future accurate expression profiling by RT-qPCR of meiosis-related genes in wheat. The two species of wheat, hexaploid bread wheat (CS) and tetraploid durum wheat (Cappelli variety) share the A and B subgenomes while D subgenome is absent in the latter. Deletion mutants for the *Ph1* locus in both hexaploid and tetraploid wheat genotypes (CS*ph1* and DES 35, respectively) have been also tested to investigate whether the presence of the *Ph1* locus might have an effect in the election of the RGs. Besides, we also studied the selected RGs in comparative analyses including somatic tissues, such as young leaves and root tips from germinating grains, which exhibit high rates of vegetative growing and cell mitosis. The results showed differences in the RGs expression stability among samples for different genotypes and tissues, hence quantitative and qualitative differences affecting the normalization were found. By addressing all these factors, our validated RGs should provide a robust normalization for the experimental conditions described in the different wheat genotypes and tissues covered by this work. The main difference lays apparently in ploidy or subgenome composition, except in meiosis samples for hexaploid wheat, in the presence and in the absence of the *Ph1* locus. *Ph1* locus seems to be affecting markedly the RGs expression stability and thus the choice for RGs, as well as increasing the number of RGs needed to normalize the CS/CS*ph1*comparisons. Curiously, the SC gene expression stability does not seem to be particularly affected by having its 5B homoeolog located within the *Ph1* locus, thus deleted in the *ph1* mutants. In fact, it is among the most recommended RGs for CS and CS*ph1*, especially in comparisons including somatic tissues.

In the original geNorm paper, the authors recommend the minimal use of the three most stable RGs in order to calculate the normalization factor in any given experiment, and a stepwise inclusion of more genes until no further significant contribution of the (n + 1)^th^ gene is observed^3^. In most published studies, the recommended number of RGs resulting from the pairwise variation (V) calculations varies from only two up to several depending on the experimental setup. However, it may be convenient sometimes the use of fewer RGs than the recommended number to keep the experimental procedures affordable. Therefore, it is not rare to find published gene quantification data obtained using only three or less RGs. Although the proposed V cutoff value of 0.15 must not be taken too strictly, according the geNorm manual, any reduction in the recommended number of RGs should be evaluated carefully and specifically for each experiment. For our validated RGs, the study performed using the *TaRec8* gene as example showed that a stepwise reduction of the RGs used for normalization might result in significant differences in data, causing loss of accuracy in gene quantification and misinterpretation of the results. We cannot recommend a minimal number of RGs for every comparison covered by this work, because we cannot assure that it will be valid for any gene of interest under study or unknown experimental variations. We suggest a recommended number of RGs that should be tested for a stepwise reduction following the ranking order, in order to experimentally determine if the use of less RGs might affect significantly the results using the appropriate statistical tests.

In conclusion, we have presented sets of validated RGs, suitable for accurate RT-qPCR normalization in wheat anthers during meiosis, as well as comparative studies with somatic tissues. RGs have been ranked accordingly to their stability in different experimental setups. This work provides a solid basis for future gene expression studies during meiosis in wheat by RT-qPCR to unravel the genetic regulation of this major biological process.

## Supplementary information

Supplementary figure.

Supplementary information.

## Data Availability

The datasets generated during and/or analysed during the current study are available from the corresponding author on reasonable request.
