# Supplementary material for: Identification and validation of reference genes for RT-qPCR normalization in wheat meiosis
Source: Sci Rep. 2020 Feb 17;10:2726. doi: 10.1038/s41598-020-59580-5 (PMC7026057; doi:10.1038/s41598-020-59580-5)
Supplement: Supplementary file 1 — Supplementary figure. [file 41598_2020_59580_MOESM1_ESM.pdf]

Supplementary Information

Identification and validation of reference genes for RT-qPCR normalization in wheat meiosis.

José Garrido[1], Miguel Aguilar[2], Pilar Prieto[1*]

[1]Plant Breeding Department, Institute for Sustainable Agriculture, Agencia Estatal Consejo Superior de Investigaciones Científicas (CSIC), Alameda del Obispo s/n, Apartado 4084, 14080 Córdoba, Spain.

[2]Área de Fisiología Vegetal. Universidad de Córdoba. Campus de Rabanales, edif. C4, 3ª planta. Córdoba, Spain.

*** Correspondence:**

pilar.prieto@ias.csic.es

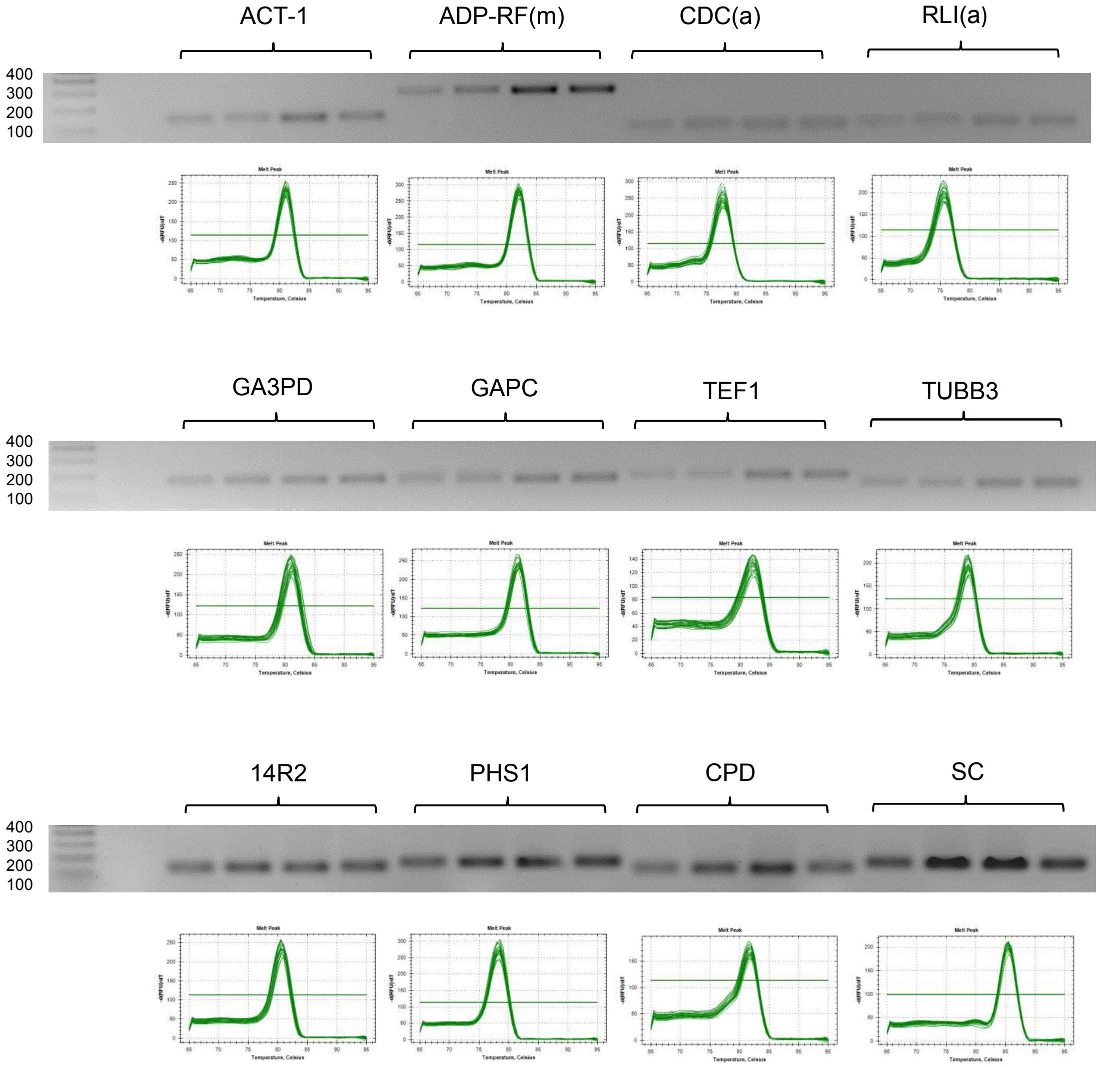

Supplementary Figure S1. Reference gene amplicon sizes (1.5% agarose gel) in wheat genotypes: CS, CS*ph1*, Cappelli and DES35 (from left to right). Melting profiles for CS are shown below the gel images as examples.
